# Simultaneous Heart-Kidney Transplant—Does Hospital Experience With Heart Transplant or Kidney Transplant Have a Greater Impact on Patient Outcomes?

**DOI:** 10.3389/ti.2023.10854

**Published:** 2023-04-05

**Authors:** Michael A. Catalano, Stevan Pupovac, Kenar D. Jhaveri, Gerin R. Stevens, Alan R. Hartman, Pey-Jen Yu

**Affiliations:** ^1^ Division of Cardiac Surgery, Department of Surgery, Hospital of the University of Pennsylvania, Philadelphia, PA, United States; ^2^ Department of Cardiovascular and Thoracic Surgery, Zucker School of Medicine at Hofstra/Northwell, Hempstead, NY, United States; ^3^ Division of Kidney Diseases and Hypertension, Department of Medicine, Zucker School of Medicine at Hofstra/Northwell, Hempstead, NY, United States; ^4^ Department of Cardiology, Zucker School of Medicine at Hofstra/Northwell, Hempstead, NY, United States

**Keywords:** outcomes, kidney transplant, heart transplant, cardiac function, volume

## Abstract

High institutional transplant volume is associated with improved outcomes in isolated heart and kidney transplant. The aim of this study was to assess trends and outcomes of simultaneous heart-kidney transplant (SHKT) nationally, as well as the impact of institutional heart and kidney transplant volume on survival. All adult patients who underwent SHKT between 2005–2019 were identified using the United Network for Organ Sharing (UNOS) database. Annual institutional volumes in single organ transplant were determined. Univariate and multivariable analyses were conducted to assess the impact of demographics, comorbidities, and institutional transplant volumes on 1-year survival. 1564 SHKT were identified, increasing from 54 in 2005 to 221 in 2019. In centers performing SHKT, median annual heart transplant volume was 35.0 (IQR 24.0–56.0) and median annual kidney transplant volume was 166.0 (IQR 89.5–224.0). One-year survival was 88.4%. In multivariable analysis, increasing heart transplant volume, but not kidney transplant volume, was associated with improved 1-year survival. Increasing donor age, dialysis requirement, ischemic times, and bilirubin were also independently associated with reduced 1-year survival. Based on this data, high-volume heart transplant centers may be better equipped with managing SHKT patients than high-volume kidney transplant centers.

## Graphical Abstract

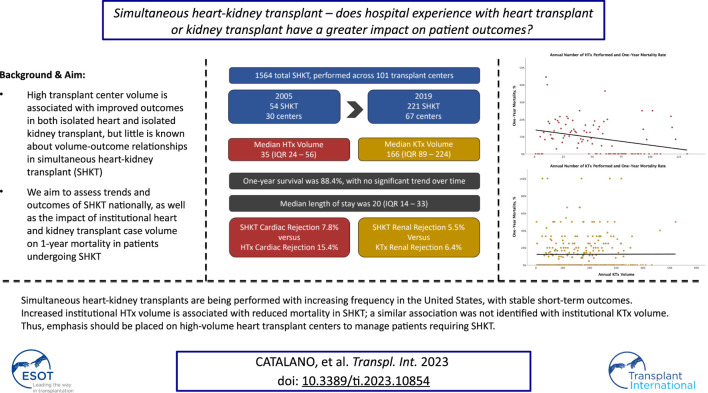

## Introduction

Kidney disease and heart disease share common risk factors. Given these shared risk factors, as well as the renal impairment with abnormal hemodynamics associated with heart failure, end-stage heart and kidney disease frequently coexist. For that reason, as well as general overall improvement in organ transplant outcomes, there has been an increase in simultaneous heart-kidney transplant (SHKT) in the United States (1, 2). Small, single-center studies have demonstrated acceptable outcomes for this procedure (3–6), and large, national database studies have revealed improved outcomes relative to isolated heart transplant (HTx) in certain patient populations (1, 7–10). While a number of ethical and clinical questions remain regarding the utilization of SHKT (2, 11), its increasing utilization in the United States warrants further study. Specifically, it is important to assess which institutions may be best suited to care for this unique patient population.

Across surgical subspecialties, institutional experience with surgical procedures is associated with significantly improved clinical outcomes (12–15). This relationship has been demonstrated in both isolated HTx (16–23) and isolated kidney transplant (KTx) (24–28), as well as in lung and liver transplants (24, 29–32). However, little is known about the relationship between surgical volume and outcomes in SHKT.

The aim of this study was to evaluate contemporary trends and outcomes of SHKT nationally and to assess the impact of institutional HTx and KTx case volume on 1-year survival in patients undergoing SHKT.

## Materials and Methods

A retrospective analysis of the United Network for Organ Sharing (UNOS) Standard Transplant Analysis and Research (STAR) files was conducted for the years 2005–2019. This study was deemed exempt from review by an Institutional Review Board as the data provided by UNOS contains no patient identifiers.

In order to understand national trends in transplant volume, we first analyzed the total volume of isolated HTx, isolated KTx, and SHKT in adult patients (≥18 years old) performed in the United States each year. In order to avoid double-counting, SHKT patients were not included in our volume analysis of isolated HTx and KTx.

All adult patients who underwent SHKT were included in our analysis; patients undergoing sequential heart-kidney transplant were excluded. Patient-specific information collected included sex, age at transplant, body mass index (BMI), diabetes, total bilirubin at transplant, creatinine at transplant, and dialysis requirement at listing (as well as an indicator of hemodialysis *versus* peritoneal dialysis) and at transplant. Dialysis requirement was selected as the indicator of renal function to allow for more consistent comparison between patients—creatinine or eGFR measurements may vary significantly based on when drawn. The utilization of cardiovascular support at time of transplant, including extracorporeal membrane oxygenation (ECMO), intraaortic balloon pump (IABP), left-ventricular assist device (LVAD), and inotropic agents was also collected. These variables were utilized as primary indicators of global hemodynamic compromise. Additionally, hemodynamics at time of transplant—including cardiac output, pulmonary artery pressures, and pulmonary capillary wedge pressures—were assessed; however, the use of quantitative measures of hemodynamics is limited given the possibility of transient fluctuations in these markers that may misrepresent the true overall hemodynamic picture based on when they were captured. Other variables included total days on waitlist, cardiac and renal ischemia time in hours, and age of heart donor.

Institutional experience in isolated heart transplant (HTx), isolated kidney transplant (KTx), and SHKT was assessed as the annual institutional transplant volume, by year. Thus, each institution is assigned a value for HTx volume, KTx volume, and SHKT volume for each year it participated in the dataset. This methodology was used in order to account for the dynamic changes in institutional experience over time, especially those that have recently opened and demonstrated rapid growth.

The primary outcome of interest was 1-year post-transplant survival. Secondary endpoints included length of stay, acute heart transplant rejection episodes requiring treatment within 1 year of transplant, and acute kidney rejection transplant episodes requiring treatment within 1 year of transplant. Length of stay was evaluated as the number of days from transplant to discharge or death. In evaluating 1-year post-transplant survival and rejection episodes requiring treatment, patients undergoing transplant in 2019 were excluded. This step was taken to avoid potential effects on survival of the COVID-19 pandemic in the year 2020.

In order to describe overall trends in utilization, the entire dataset was queried to identify all HTx and KTx over the selected timeframe, as well as changes over time. Trends were also assessed among the selected sample of SHKT. Next, descriptive analysis was conducted for the selected sample, including patient demographics, donor demographics, risk factors, organ ischemia time, and institutional experience. Each of these factors was also assessed as a predictor of 1-year survival in univariate and multivariable analysis. In univariate analysis, the Pearson chi-square test was used to analyze categorical variables, and Student’s t-test was used to evaluate continuous variables. In multivariable analysis, binary logistic regressions were conducted, and odds-ratios (OR) and p-values are reported. Multivariable analysis was also conducted to assess predictors of secondary endpoints. Length of stay was assessed using multivariable linear regression, with coefficients and p-values reported. Acute transplant rejection episodes were assessed using binary logistic regression, with OR and p-values reported.

All statistical analyses were performed using SAS, version 9.4 (SAS Institute, Inc., Cary, NC). All P-values were 2-sided with a significance threshold of <0.05. A 95% confidence interval (*p* < 0.05) was defined as statistical significance for all analyses.

## Results

**FIGURE 1:**
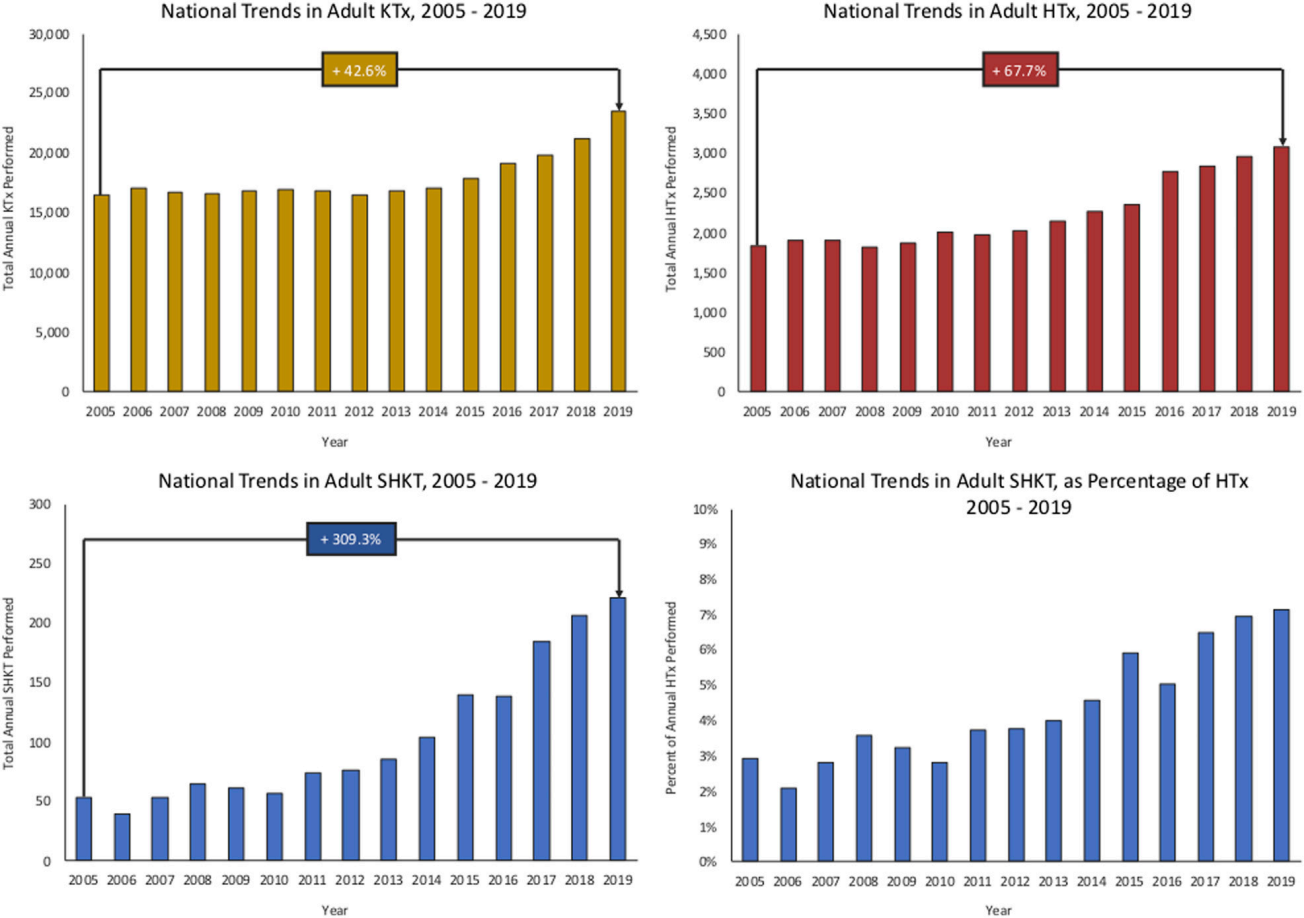
National trends in adult isolated KTx, HTx, SHKT, and SHKT as a proportion of total HTx (2005–2019). HTx, heart transplant; KTx, kidney transplant; SHKT, simultaneous heart-kidney transplant.

Trends in utilization of SHKT, HTx, and KTx are presented in Figure 1. Over the study period of 2005–2019, we identified 1564 SHKT, increasing from 54 procedures performed across 30 centers in 2005 to 221 procedures across 67 centers in 2019 (309.3% volume growth). While incidence of isolated HTx (1,841 in 2005, to 3,088 in 2019, 67.7% volume growth) and isolated KTx (16,489 in 2005, to 23,510 in 2019, 42.6% volume growth) also increased over the study period, the magnitude of growth was substantially lower. Utilization of SHKT increased from 2.9% of all heart transplants performed in 2005, to 7.2% in 2019. We observed a 1-year mortality of 11.5% for SHKT, with no significant change over time. Median length of stay was 20.0 days (IQR 14.0–33.0). Cardiac rejection episodes in the first-year post-transplant occurred in 7.8% of SHKT patients (*versus* 15.4% of isolated HTx), and kidney allograft rejection episodes in the first-year post-transplant occurred in 5.5% of SHKT patients (*versus* 6.4% of isolated KTx).

Baseline characteristics for patients undergoing SHKT and institutional transplant volume, and their association with 1-year survival, for the years 2005–2018, are presented in Table 1. Across the 1,343 patients, mean recipient age was 54.1 ± 11.5 years; mean donor age was 31.7 ± 11.4 years. Male patients made up 79.1% of the sample. There was no significant association between recipient age or sex and survival in univariate analysis; increasing donor age was associated with decreased survival (*p* = 0.019). Dialysis requirement was observed in 30.0% of patients at listing (including 27.0% of patients on hemodialysis and 3.0% of patients on peritoneal dialysis) and 38.2% of patients at time of transplant. Hemodialysis at listing trended towards an association with reduced survival (*p* = 0.076); any dialysis at transplant was associated with decreased survival (*p* < 0.001). Other patient and transplant factors associated with decreased survival on univariate analysis included elevated total bilirubin (*p* < .001), increased cardiac ischemia time (*p* = 0.007), and increased renal ischemia time (*p* = 0.046).

**TABLE 1:** Baseline characteristics as predictors of survival (2005–2018).

Variable	Total	Died	Survived	P-value
Total (%)	1,343	155 (11.5)	1,188 (88.5)	
Male Sex	1,062 (79.1)	123 (79.4)	939 (79.0)	0.927
Recipient Age, years	54.1 ± 11.5	54.3 ± 11.2	54.0 ± 11.6	0.763
Donor Age, years	31.7 ± 11.4	33.7 ± 11.5	31.4 ± 11.4	0.019
Recipient BMI	26.6 ± 4.9	27.3 ± 5.4	26.5 ± 4.8	0.062
Hemodynamics at Transplant				
Cardiac Output	4.9 ± 1.7	5.0 ± 1.8	4.9 ± 1.6	0.792
PA Systolic Pressure	43.9 ± 13.8	47.0 ± 13.6	43.5 ± 13.8	0.003
PA Diastolic Pressure	21.3 ± 7.9	23.3 ± 7.6	21.0 ± 7.9	0.001
Mean PA Pressure	29.9 ± 9.5	32.3 ± 9.1	29.6 ± 9.5	0.002
PCWP	19.8 ± 8.4	21.3 ± 7.7	19.6 ± 8.4	0.028
Dialysis at Listing				
Hemodialysis	362 (27.0)	51 (32.9)	311 (26.2)	0.076
Peritoneal Dialysis	40 (3.0)	6 (3.9)	34 (2.9)	0.487
Dialysis at Transplant	513 (38.2)	83 (53.5)	430 (36.2)	<0.001
Creatinine at Transplant	3.5 ± 2.6	4.0 ± 3.1	3.4 ± 2.5	0.019
Total Bilirubin, mg/dL	1.2 ± 3.4	2.1 ± 6.7	1.1 ± 2.7	<0.001
Waiting List Days	219.5 ± 351.9	199.3 ± 287.2	222.1 ± 359.6	0.448
Recipient Diabetes	580 (43.2)	72 (46.5)	508 (42.8)	0.382
ECMO at Transplant	17 (1.3)	4 (2.6)	13 (1.1)	0.119
IABP at Transplant	109 (8.1)	17 (11.0)	92 (7.7)	0.166
Inotropes at Transplant	603 (44.9)	61 (39.4)	542 (45.6)	0.140
LVAD at Transplant	275 (20.5)	30 (19.4)	245 (20.6)	0.712
Cardiac Ischemic Time, hours	3.1 ± 1.0	3.3 ± 1.1	3.1 ± 1.0	0.007
Kidney Ischemic Time, hours	14.6 ± 8.2	15.9 ± 8.5	14.4 ± 8.2	0.046
Annual HTx Volume	43.3 ± 29.8	36.4 ± 24.2	44.2 ± 30.4	0.002
Annual KTx Volume	162.8 ± 92.1	152.0 ± 90.4	164.2 ± 92.3	0.121

Pearson chi-square test was used for evaluation of categorical variables, with column percent in parentheses; Student’s t-test was used for evaluation of continuous variables.

BMI, body mass index; PA, pulmonary artery; PCWP, pulmonary capillary wedge pressure; ECMO, extracorporeal membrane oxygenation; HTx, heart transplant; IABP, intraaortic balloon pump; KTx, kidney transplant; LVAD, left ventricular asssit device.

At the time of transplant, 603 (44.9%) patients were supported by inotropes, 275 (20.5%) were supported by an LVAD, 109 (8.1%) were supported by an IABP, and 17 (1.3%) were supported by ECMO. Utilization of inotropic or mechanical circulatory support was not associated with 1-year survival. While there was no significant association between mechanical circulatory support and survival, elevated pulmonary artery pressures and pulmonary capillary wedge pressures were associated with reduced 1-year survival (Table 1).

**FIGURE 2:**
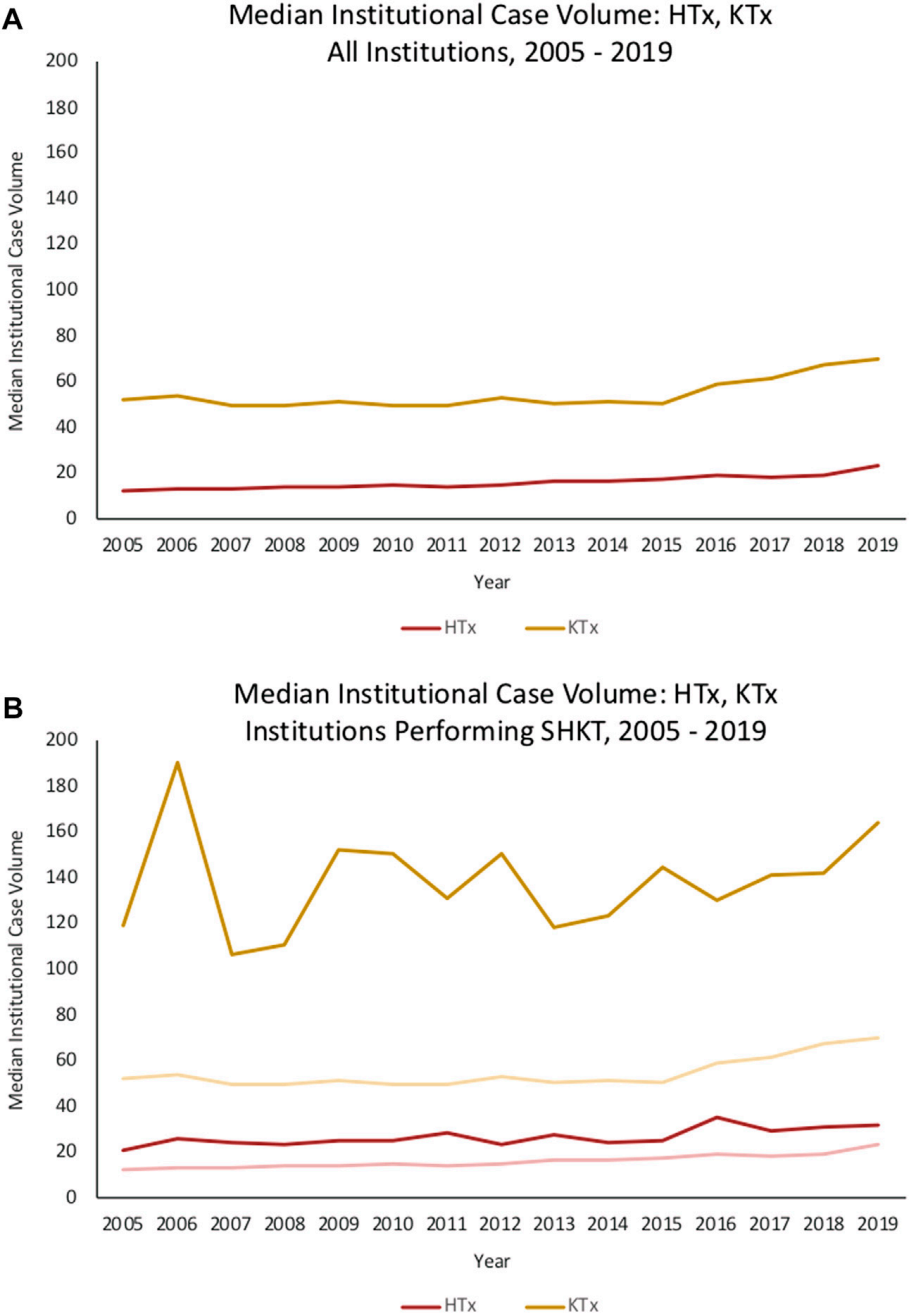
Trends in median institutional case volume for HTx, KTx, and SHKT among **(A)** all institutions in the United States, 2005–2019, and **(B)** only institutions performing SHKT in the United States, 2005–2019. HTx, heart transplant; KTx, kidney transplant; SHKT, simultaneous heart-kidney transplant.

Median annual institutional HTx volume across the sample of institutions performing SHKT was 35.0 (IQR 24.0–56.0); median annual institutional KTx volume was 166.0 (IQR 89.5–224.0). Centers performing SHKT had greater annual experience with isolated HTx and KTx than centers which did not perform SHKT (Figure 2). In 2019, median HTx volume across all institutions was 23, compared to median HTx volume of 32 across institutions performing SHKT. Similarly, median KTx volume across all institutions was 70, compared to median KTx volume of 164 across institutions performing SHKT. On univariate analysis, transplant centers performing a higher volume of annual heart transplants had improved 1-year survial in their SHKT patients (annual volume of 44.2 ± 30.4 in patients who survived, vs. annual volume of 36.4 ± 24.2 in patients who died, *p* = 0.002). There was no significant association between annual kidney transplant volume and survival (*p* = 0.121) (Table 1).

Multivariable analysis of factors associated with 1-year suvival in SHKT patients is shown in Table 2. Increased annual heart transplant volume remained associated with improved 1-year survival (OR 1.12 for every 10 heart transplants, *p* = 0.004). Other factors associated with decreased 1-year survival included increasing donor age, increasing recipient serum bilirubin, dialysis requirement at transplant, and increasing cardiac ischemia time. Annual kidney transplant volume was not associated with 1-year survival (*p* = 0.485).

**TABLE 2:** Multivariable predictors of 1-year survival in SHKT (2005–2018).

Variable	Odds ratio for mortality (95% CI)	p-value
Annual Heart Transplant Volume (+10)	1.12 (1.04–1.21)	0.004
Annual Kidney Transplant Volume (+10)	1.01 (0.99–1.03)	0.485
Recipient Male Sex	1.19 (0.75–1.88)	0.458
Recipient Age (+10)	0.89 (0.74–1.06)	0.221
Donor Age (+10)	0.83 (0.70–0.98)	0.031
Recipient BMI (+5)	0.87 (0.71–1.06)	0.187
Dialysis at Transplant	0.46 (0.31–0.68)	<0.001
Recipient Serum Bilirubin (+0.3)	0.93 (0.90–0.97)	<0.001
Total Days on Waiting List (+30)	1.01 (0.99–1.02)	0.537
Recipient Diabetes	1.08 (0.72–1.61)	0.690
ECMO at Transplant	0.46 (0.13–1.61)	0.228
Intraaortic Balloon Pump at Transplant	0.71 (0.38–1.33)	0.292
Inotropes at Transplant	1.19 (1.78–1.78)	0.370
Left Ventricular Assist Device at Transplant	1.17 (0.71–1.92)	0.534
Cardiac Ischemia Time (+1 h)	0.78 (0.66–0.92)	0.004
Kidney Ischemia Time (+10 h)	0.83 (0.68–1.02)	0.083

BMI, body mass index; ECMO, extracorporeal membrane oxygenation; SHKT, simultaneous heart-kindey transplant.

Factors associated with prolonged length of stay after transplant in multivariable analysis included younger transplant recipient age, older heart donor age, higher recipient bilirubin, and longer renal ischemia time (Table 3). None of the assessed variables were associated with 1-year cardiac rejection episodes in multivariable analysis (Table 3). The presence of dialysis at transplant and reduced cardiac ischemia time was associated with increased risk of 1-year renal rejection episodes (Table 3).

**TABLE 3:** Multivariable predictors of LOS, 1-year HTx rejection, and 1-year KTx rejection (2005–2018).

	LOS^a^		HTx rejection		KTx rejection	
Variable	Coefficient	P	OR	P	OR	P
Annual HTx Volume (+10)	−0.5 (−1.2, 0.1)	0.119	0.95 (0.88, 1.03)	0.210	0.94 (0.85, 1.04)	0.210
Annual KTx Volume (+10)	−0.2 (−0.4, 0.0)	0.055	1.01 (0.99, 1.04)	0.239	1.01 (0.99, 1.04)	0.339
Recipient Male Sex	2.6 (−2.2, 7.3)	0.283	1.09 (0.64, 1.86)	0.750	0.96 (0.52, 1.77)	0.897
Recipient Age (+10)	−2.4 (−4.1, −0.7)	0.007	0.89 (0.74, 1.08)	0.246	0.83 (0.67, 1.04)	0.106
Donor Age (+10)	2.2 (0.4, 3.9)	0.014	1.01 (0.83, 1.22)	0.912	0.97 (0.77, 1.22)	0.793
Recipient BMI (+5)	0.2 (−1.8, 2.2)	0.828	0.92 (0.73, 1.16)	0.474	1.09 (0.84, 1.42)	0.506
Dialysis at Transplant	2.6 (−1.4, 6.6)	0.208	0.99 (0.64, 1.55)	0.976	2.19 (1.32, 3.65)	0.003
Recipient Bilirubin (+0.3)	0.6 (0.2, 1.1)	0.003	1.00 (0.96, 1.05)	0.878	1.00 (0.95, 1.05)	0.993
Days on Waiting List (+30)	−0.1 (−0.3, 0.1)	0.246	1.00 (0.98, 1.02)	0.937	1.02 (1.00, 1.03)	0.082
Recipient Diabetes	3.0 (−1.0, 7.0)	0.141	1.04 (0.66, 1.64)	0.864	1.18 (0.70, 2.02)	0.534
ECMO at Transplant	11.9 (−1.4, 25.3)	0.080	0.70 (0.09, 5.45)	0.733	0.98 (0.12, 7.95)	0.988
IABP at Transplant	−0.5 (−6.4, 5.5)	0.879	0.86 (0.38, 1.93)	0.712	0.57 (0.17, 1.87)	0.353
Inotropes at Transplant	−3.1 (−7.1, 0.9)	0.129	1.11 (0.71, 1.73)	0.646	1.58 (0.93, 2.66)	0.090
LVAD at Transplant	−1.5 (−6.7, 3.8)	0.581	0.79 (0.43, 1.45)	0.450	0.88 (0.43, 1.77)	0.711
HTx Ischemia (+1 h)	−0.5 (−2.4, 1.3)	0.582	1.00 (0.82, 1.22)	0.978	0.73 (0.56, 0.94)	0.016
KTx Ischemia (+10 h)	4.4 (2.1, 6.7)	<0.001	1.09 (0.86, 1.39)	0.461	0.78 (0.56, 1.10)	0.154

^a^ LOS analysis includes patients undergoing SHKT in 2005–2019; 1-year rejection episode analysis includes patients undergoing SHKT in 2005–2018.

BMI, body mass index; ECMO, extracorporeal membrane oxygenation; HTx, heart transplant; IABP, intraaortic balloon pump; KTx, kidney transplant; LOS, length of stay; LVAD, left ventricular asssit device; OR, odds ratio.

## Discussion

Our study provides a contemporary assessment of the utilization and outcomes of SHKT, and is the first to assess the impact of institutional experience with HTx and KTx on SHKT outcomes. We identify a continued trend of increased SHKT utilization, increasing 309.3% over 14 years. We also observe a significant association between annual institutional HTx volume and 1-year survival in SHKT patients. A similar association between institutional KTx volume and SHKT outcomes was not observed. Further, we found that dialysis at transplant, increased donor age, increased bilirubin, and prolonged cardiac ischemia time are independently associated with reduced 1-year survival.

Our finding of increased utilization of SHKT, out-of-proportion to the increase in isolated HTx, is consistent with prior studies of SHKT in the United States. Karamlou et al., who assessed SHKT vs. isolated HTx in the United States from 2000–2010, found that national HTx volume increased 3.6% over time, while prevalence of SHKT increased 147% (1). Similarly, Melvinsdottir et al. found that, while staged heart-kidney transplant utilization has decreased from 1990–2016, SHKT utilization has increased (2). We demonstrate that this trend has continued, as SHKT as a proportion of total HTx has increased from 2.9% in 2005% to 7.2% in 2019. The increase in utilization has likely been influenced by evolving literature demonstrating acceptable outcomes of patients undergoing SHKT. In 1997, Laufer et al. retrospectively assessed the clinical and immunologic outcomes of six patients who underwent SHKT at their institution. With a mean follow-up of 32 months, they identified 100% survival, with no episodes of renal transplant rejection. Further, in a comparison to isolated HTx patients, there was no difference in rates of cardiac rejection (5). Hermsen et al., similarly, reviewed patient and graft survival across 19 SHKTs performed at their institution from 1987–2006, comparing outcomes to isolated HTx, isolated KTx, and staged heart-kidney transplant. They found no difference in patient or graft survival; further, they identified reduced rates of coronary allograft vasculopathy and increased time to graft rejection episodes in SHKT patients, suggesting an immunologic benefit to simultaneous organ transplantation (4). Our finding of reduced cardiac and kidney allograft rejection episodes for SHKT patients, as compared with isolated HTx and KTx, supports this suggested immunologic benefit. Grupper et al., in their 2017 study of 35 SHKT patients, identified survival rates of 97% at 6 months, 91% at 1 year, and 86% at 3 years (3). This 1-year mortality rate of 9% is comparable to our finding of 11.5% 1-year mortality nationally.

As utilization of SHKT continues to increase nationally, it is vital to understand if there are centers that may be better suited to care for this unique patient population. Based on the existence of a volume-outcome relationship in organ transplantation (16–32) and other surgical fields (12–15), our focus was on identifying whether experience with one or both components of this particular multi-organ transplant has an impact on outcomes. Our finding that increased annual HTx volume is associated with improved SHKT survival is consistent with our hypothesis of the existence of a volume-outcome relationship, and it is consistent with prior isolated HTx literature. In their study of isolated HTx in Korea, Nam et al. assessed outcomes in 833 adult transplants across 17 centers, identifying in-hospital mortality of 3.7% in high-volume centers (>20 HTx/year), 10.1% in medium-volume centers (10–20 HTx/year), and 18.6% in low-volume centers (<10 HTx/year). This difference persisted in evaluation of 10-year survival (19). Differences in short-term and long-term HTx patient and graft survival have also been demonstrated using UNOS in both congenital (17, 18) and general adult populations (16, 21–23). In order to understand why a volume-outcome relationship may exist in HTx, Arnaoutakis et al. assessed institutional volume as an effect modifier on the relationship between patient risk and survival. In their analysis, low-volume centers (<7 HTx/year) had increased mortality relative to medium-volume (7–15 HTx/year) and high-volume (>15 HTx/year) centers. However, the difference in mortality was primarily driven by outcomes in high-risk patients; the effect of center volume on outcomes in low-risk patients is minimal (16). This suggests that institutional experience in HTx may primarily play a role in caring for sicker, more complex patients. While we do not quantify risk in our study, SHKT patients tend to carry a greater burden of comorbidities than isolated HTx patients, potentially explaining why a volume-outcome relationship was observed. It is, indeed, possible that lower volume centers included in our sample were transplanting sicker patients; however, despite including comorbidities in our multivariable analysis, case volume remained a significant predictor of post-operative survival, suggesting that experience may be important across all risk groups. Another study that provides insight into the reason that experience in transplant affects outcomes is that by Kilic et al. In their study of isolated lung transplant, they found no association between center volume and occurrence of major post-operative complications. However, they found that in patients who do experience complications, risk of mortality is significantly greater at low-volume centers (29). This, similar to the results of our study, suggests that higher-volume institutions are better equipped to care for the most complex transplant patients.

In contrast to the HTx volume-outcome relationship, we observed no association between institutional isolated KTx experience and SHKT outcomes. This may be rationalized by the difference in expected short-term mortality in isolated HTx *versus* isolated KTx—given the substantially greater risk associated with the HTx component of the simultaneous procedure, it can be expected that strong experience with HTx drives outcomes in SHKT. Moreover, center selection bias may play a role. While median annual KTx volume across all institutions in the United States during our study period is approximately 60 KTx/year, the median annual KTx volume among the subset of institutions performing SHKT is 166 KTx/year. Thus, we are already selecting for relatively high-volume KTx institutions, which may explain why differences in volume have less of an impact on outcomes in our select population. The existing literature in isolated KTx also less consistently demonstrates the volume-outcome relationship observed in isolated HTx (28). Axelrod et al. identify a significantly increased risk of mortality and 1-year renal graft loss in isolated KTx at low-volume centers as compared to high-volume centers. On the other hand, Sonnenberg et al. found no association between KTx volume quartile (ranging from Q1 <66 KTx to Q4 >196 KTx) and 3-year graft or patient survival (33).

While we identified a volume-outcome relationship in patient survival, the same relationship was not observed between transplant center experience and 1-year cardiac and renal allograft rejection episodes. Interestingly, however, we did identify a higher rate of cardiac allograft rejection compared to renal allograft rejection among the population of SHKT patients (7.8% *versus* 5.5%); while it is challenging to ascertain the cause of this difference, one likely explanation is the difference in identification of rejection episodes—while renal allograft may only be identified when clinical signs present, planned endomyocardial biopsies allow for the detection of subclinical rejection episodes. Another interesting finding in multivariable analysis was the significant association between cardiac ischemic time and renal allograft rejection, with prolonged cardiac ischemic time associated with lower rates of renal allograft rejection. Without knowing exactly when each renal allograft implantation began relative to cardiac allograft implantation, this is challenging to explain. However, a common critique of SHKT is that the hemodynamic instability and coagulopathy that occur immediately during and after heart transplant place the renal allograft at significant risk of dysfunction and early rejection. Thus, some advocate for a short period of hemodynamic recovery in the operating room prior to initiation of the renal allograft transplantation. It is, therefore, possible that reduced cardiac allograft ischemic time is associated with a more rapidly performed procedure overall, including rapid renal allograft implantation, greater early exposure of the renal allograft to hemodynamic instability, and greater risk of renal allograft compromise and early graft rejection.

In addition to understanding volume-outcome relationships, we also sought to identify comorbidities associated with 1-year survival. We found that dialysis-dependent patients undergoing SHKT have decreased 1-year survival and increased rates of renal allograft rejection relative to patients not requiring pre-transplant dialysis. Despite the increased risk identified, there is substantial literature that suggests that SHKT provides benefit relative to isolated HTx in patients with the most severe degrees of kidney dysfunction. For instance, Karamlou et al. compared 593 SHKT and 26,183 isolated HTx, assessing the impact of pre-operative renal function on benefit of SHKT relative to isolated HTx. They observed similar overall survival; however, when stratifying by eGFR quintiles, patients in the lowest quintile (eGFR <37 mL/min) undergoing isolated HTx had significantly worse survival than patients undergoing SHKT, suggesting a relative benefit of SHKT (1). The utilization of eGFR as a measure of renal function in UNOS studies is limited by the fact that it is based on a single creatinine measure, often that most proximal to the transplant date. Thus, other studies have attempted to expand upon the association between renal function and SHKT benefit by looking specifically at dialysis-dependence. Gill et al. assessed clinical outcomes in 263 SHKT patients relative to isolated HTx. Overall adjusted risk of death was found to be 44% lower with SHKT compared to isolated HTx, and this difference was driven by dialysis-dependent patients (8). Schaffer et al. compared outcomes of SHKT *versus* isolated HTx in patients with eGFR <50 mL/min, stratified by dialysis-dependence. Five-year posttransplant survival was improved in SHKT patients among dialysis-dependent patients (73% vs. 51%) as well as those with non-dialysis-dependent renal insufficiency (80% vs. 69%) (10). While kidney recovery for patients with non-dialysis-dependent renal insufficiency is possible following isolated HTx, these findings suggest that SHKT may provide a significant survival advantage in this patient population. Thus, while our results highlight that dialysis-dependence represents an independent risk factor for poor outcomes among SHKT patients, there exists strong evidence that SHKT remains beneficial as compared to isolated HTx in dialysis-dependent patients.

Our study is not without limitations. First, this is a retrospective study using a clinical database with inherent limitations. In the evaluation of a clinically complex patient population, nuances in pathology and management may not be captured by the database. Second, our study does not provide insight into why volume-outcome relationships are observed in SHKT. While we identify increased ischemic time as a predictor of decreased survival and high-volume centers are likely to have reduced ischemic times, further explanation is an important area of future study. Third, we do not include sequential heart-kidney transplant patients in our analysis; this is because the volume of sequential heart-kidney transplant is quite low in the United States, the patients undergoing sequential heart-kidney transplant are inherently different than SHKT patients (2), and this patient population has already been quite well described (2). Melvinsdottir et al. identify that sequential heart-kidney transplant may have improved outcomes relative to SHKT; however, they also show that sequential heart-kidney transplant volume in the United States is falling out of favor, with only 6 procedures performed in 2016 (2).

In summary, simultaneous heart-kidney transplants are being performed with increasing frequency in the United States, with stable short-term outcomes. Increased institutional HTx volume, but not KTx volume, is associated with improved 1-year survival in SHKT. Thus, emphasis should be placed on high-volume heart transplant centers to manage patients requiring SHKT.

## Data Availability

Publicly available datasets were analyzed in this study, available through the United Network for Organ Sharing (UNOS) and the Organ Procurement and Transplantation Network (OPTN), and they can be found here: https://optn.transplant.hrsa.gov/data/view-data-reports/request-data/.

## Abbreviations

ECMO, extracorporeal membrane oxygenation; HTx, heart transplant; IABP, intra-aortic balloon pump; KTx, kidney transplant; LVAD, left ventricular assist device; NDDRI, non-dialysis-dependent renal insufficiency; OR, odds-ratio; SHKT, simultaneous heart-kidney transplant; STAR, standard transplant analysis and research; UNOS, United Network for Organ Sharing.
